# Clinicopathological and prognostic significance of c-Met overexpression in breast cancer

**DOI:** 10.18632/oncotarget.18142

**Published:** 2017-05-24

**Authors:** Xixi Zhao, Jingkun Qu, Yuxin Hui, Hong Zhang, Yuchen Sun, Xu Liu, Xiaoyao Zhao, Zitong Zhao, Qian Yang, Feidi Wang, Shuqun Zhang

**Affiliations:** ^1^ Department of Oncology, The Second Affiliated Hospital of Xi’an Jiaotong University, Xi’an, Shaanxi 710004, P.R. China; ^2^ The Second Department of Thoracic Surgery, The First Affiliated Hospital of Xi’an Jiaotong University, Xi’an, Shaanxi 710061, P.R. China; ^3^ The School of Traditional Chinese Medicine, Hunan University of Chinese Medicine, Changsha, Hunan 410208, P.R. China; ^4^ The Department of Radiation Oncology, The First Affiliated Hospital of Xi’an Jiaotong University, Xi’an, Shaanxi 710061, P.R. China

**Keywords:** breast cancer, c-Met, prognosis, meta-analysis

## Abstract

**Background:**

c-Met has been shown to promote organ development and cancer progression in many cancers. However, clinicopathological and prognostic value of c-Met in breast cancer remains elusive.

**Methods:**

PubMed and EMBASE databases were searched for eligible studies. Correlation of c-Met overexpression with survival data and clinicopathological features was analyzed by using hazard ratio (HR) or odds ratio (OR) and fixed-effect or random-effect model according to heterogeneity. All statistical tests were two-sided.

**Results:**

32 studies with 8281 patients were analyzed in total. The c-Met overexpression was related to poor OS (overall survival) (HR=1.65 (1.328, 2.051)) of 18 studies with 4751 patients and poor RFS/DFS (relapse/disease free survival) (HR=1.53 (1.20, 1.95)) of 12 studies with 3598 patients. Subgroup analysis according to data source/methods/ethnicity showed c-Met overexpression was related to worse OS and RFS/DFS in Given by author group, all methods group and non-Asian group respectively. Besides, c-Met overexpression was associated with large tumor size, high histologic grade and metastasis.

**Conclusions:**

Our results showed that c-Met overexpression was connected with poor survival rates and malignant activities of cancer, including proliferation, migration and invasion, which highlighted the potential of c-Met as significant candidate biomarker to identify patients with breast cancer at high risk of tumor death.

## INTRODUCTION

Breast cancer is the most common cancer type and the second leading cause of cancer death in women worldwide and is expected to account for 29% all new cancer diagnoses for female [1]. Besides, breast cancer is a heterogeneous disease that comprises a variety of pathologies and displays a range of histological characteristics and clinical outcomes [2]. Nowadays, the focus of treatment strategies is using chemotherapy to induce cancer cell apoptosis, resistance to hormone therapy and targeted therapy. However, the prognosis of breast cancer patients remains unsatisfactory [3]. Biomarkers play an essential role in the management of patients with invasive breast cancer and may be used to predict outcome and aid adjunct therapy decision-making.

The tyrosine kinase c-Met, also called MET and hepatocyte growth factor receptor (HGFR), is a key regulator of organ development and cancer progression and has been studied in many cancer types such as lung cancer, gastric cancer, prostate cancer and so on [4–7]. c-Met inhibitors also have been tested in many cancers and shown promising results in lung cancer, ovarian cancer and so on [5, 8]. In breast cancer, previous studies have yielded mixed results. Some studies showed favorable association, some reported no significance, while some others reported a negative prognostic effect between c-Met overexpression and prognosis [9–11]. And two previously published meta-analysis with small samples yielded conflicting results of OS for breast cancer patients [12, 13]. Therefore, more systematic studies are needed to acquire high quality evidence-based results of the prognostic value of c-Met to identify patients who would benefit from c-Met targeted therapy and guide future clinical trial.

## RESULTS

### Description of included studies

**Figure 1:**
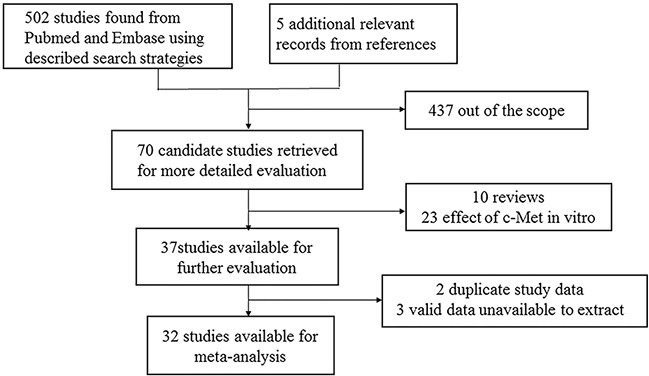
Selection of studies Flow chart showed selection of the studies in the meta-analysis.

507 records were identified in total and then 70 candidate studies were selected. Through further screening, 33 studies were excluded because of *in vitro* experiment and reviews. Among the remaining studies, three studies were performed in the same institution and only the most recent study was included. Finally, 32 studies were included and the detailed literature search and study selection could be seen in Figure 1.

There were 32 studies with 8281 patients in total involved in our meta-analysis. Thereinto, 18 studies with 4751 patients were available for OS survival data and 12 studies with 3598 patients were available for RFS/DFS survival data. There were 24 (75%) articles using immunohistochemistry method to determine the overexpression of c-Met and 8 (25%) articles using RT-PCR, FISH, RPPA and MIP respectively. All the articles included were retrospective. The study quality was assessed using the Newcastle-Ottawa quality assessment scale, generating scores ranging from 5 to 8 with a mean of 6.625 (Table 1).

**Table 1:** Characteristics of included studies

First author	Year	Patients source	Type of patients	Protein location	Age median (range)	Patients No.	Histological grade/Stage	Technique	No. of patients with protein overexpression(%)	Analysis	Follow-up years median (range)	Survival outcome	Scores of study
Ren, X.	2016	China	TNBC	membrane/cytoplasm	50.7(24-81)	127	G1-3	IHC	55(43.3%)	independent	NA	RFS/OS	7
Zagouri, F.	2014	Greece	ER+ / HER2+	membrane	57(31-82)	78	G1-3	IHC	3(3.8%)	blind	(0-14)	RFS/OS	6
Koh, Y. W.	2014	korea	invasive BC	cytoplasm	44 (20–78)	129	G1-3	IHC	89(68.9%)	independent/blind	3.2(0.7-7.5)	RFS	7
Kim, Y. J.	2014	Korea	invasive BC	membrane/cytoplasm	46(20-80)	924	I-IV	IHC	386(41.8%)	independent/blind	5.8(0-11.7)	DFS/OS	8
Inanc, M.	2014	Turkey	TNBC	membrane/cytoplasm	47(27-79)	97	G1-3	IHC	52(53.6%)	independent	NA	RFS/OS	8
Hsu, Y. H.	2014	America/China	TNBC	NA	NA	170	NA	PT-PCR	NA	NA	NA	OS	6
de Melo Gagliato, D.	2014	America	IDC	NA	47(31-72)	63	G1-3	FISH	3(4.7%)	NA	NA	OS	7
Baccelli, I.	2014	Germany	HR+/HER2-	membrane/cytoplasm	60.77(30-86)	255	G1-3	IHC	100(39%)	independent/blind	11.1	OS	7
Ho-Yen, C. M.	2014	Britain	invasive BC	cytoplasm	54(37-69)	1274	G1-3	IHC	NA	independent/blind	10.1(1.9-16.8)	OS	8
Zagouri, F.	2013	Australia/greece	TNBC	membrane	59(23-85)	170	NA	IHC	89(52%)	blind	7.4(6.5-8.3)	OS/RFS	8
Gonzalez-Angulo, A. M.	2013	America	early stage BC	NA	53(25-87)	971	G1-3	MIP	82 (8.44%)	independent/blind	7.4	RFS	8
Raghav, K. P.	2012	America	invasive BC	NA	51(23-85)	257	G1-3	RPPA	181(70.4%)	NA	3.5(0.4-23.1)	RFS/OS	8
Minuti, G.	2012	Italy/poland	HER2+ invasive BC	NA	55(33-80)	130	G2-3	FISH	36(27.7%)	NA	NA	OS	7
Gisterek, I.	2011	poland	invasive BC	NA	57(29-83)	302	G1-3	IHC	82(26.5%)	NA	NA	OS	5
Valente, G.	2009	Italy/poland	invasive BC	cytoplasm	NA	35	G1-3	IHC	28(80%)	independent	NA	NA	6
Ponzo, M. G.	2009	Canada	invasive BC	NA	54.1(42.8-65.4)	668	NA	IHC	NA	NA	3.58	RFS	5
Carracedo, A.	2009	Spain	invasive BC	NA	NA	168	NA	IHC	65(38.7%)	NA	NA	NA	5
Vendrell, J. A.	2008	Caucasian	ER+	NA	55.5(31-77)	33	G1-3	PT-PCR	17(51.5%)	NA	NA	RFS/OS	7
Pozner-Moulis, S.	2007	America	IDC	nuclear	58.1	274	G1-3	IHC	123(44.9%)	NA	12.8	OS	6
Lindemann, K.	2007	Germany	pure DCIS	membrane/cytoplasm	53.8(37.8-85.7)	39	G1-3	IHC	16(41%)	independent/blind	3.86	NA	6
Gotte, M.	2007	Germany	DCIS	membrane/cytoplasm	59(18-94)	142	NA	IHC	69(48.6%)	independent/blind	NA	NA	6
Chen, H. H.	2007	China	T1–2 N0 M0	membrane/cytoplasm	50(25-75)	104	G1-3	IHC	65(63.1%)	independent/blind	3.8 (0.8-13.5)	DFS	7
Garcia, S.	2007	France	IDC	cytoplasm	54.2(31-84)	916	G1-3	IHC	320(34.9%)	NA	6.5(4-10)	NA	6
Chen, C. C.	2006	China	NA	NA	NA	102	G1-3	PT-PCR	45(44%)	NA	NA	NA	7
Lengyel, E.	2005	Germany	lymph node +	membrane/cytoplasm	54(28-80)	40	NA	IHC	12(30%)	independent/blind	5.8(1-10.2)	DFS	6
Tolgay Ocal, I.	2003	America	lymph node -	cytoplasm	NA	324	G1-3	IHC	71(22%)	independent/blind	14.3(0.3-53.8)	OS	7
Greenberg, R.	2003	Israel	IDC	NA	58(42-74)	31	G1-3	PT-PCR	23(74.2%)	NA	NA	NA	6
Edakuni, G.	2001	Japan	IDC	membrane/cytoplasm	51(30-88)	88	G1-3	IHC	40(45.5%)	NA	4.4(0.2-16.1)	NA	6
Nakopoulou, L.	2000	Greece	invasive BC	cytoplasm	57(28-84)	69	G1-3	IHC	40(58%)	independent	5.8(5-8)	OS	7
Camp, R. L.	1999	America	IDC	NA	50.9(32-84)	113	G1-3	IHC	28(25%)	independent/blind	4.2(0-5)	OS	7
Ghoussoub, R. A.	1998	America	IDC	cytoplasm	58.1(26-88)	91	G1-3	IHC	20(22%)	independent/blind	5.1(0.1-14.1)	OS	7
Narita, T.	1997	Japan	NA	NA	NA	97	NA	IHC	48(49.5%)	NA	NA	NA	5

BC: breast cancer; TNBC: triple negative breast cancer; OS: overall survival; RFS/DFS: relapse/disease free survival; IDC: invasive ductal carcinoma; DCIS: ductal carcinoma in situ; ER: estrogen receptor; PR: progestogen receptor; HER-2: human epidermal growth factor 2; IHC: immunohistochemistry; RT-PCR: real-time quantitative PCR; RPPA: reverse phase protein lysate microarray; FISH: fluorescence in situ hybridization; MIP: molecular inversion probes; NA: not available.

### Data synthesis: clinicopathological features

Our results showed that c-Met overexpression was significantly correlated to large tumor size, OR=1.785 (1.480, 2.153); high histologic grade, OR=1.547 (1.108, 2.158) and distant metastasis, OR=20.431 (1.869, 223.360). However, high c-Met overexpression was not found to be associated with Menopausal status, OR=0.758 (0.529, 1.086); age, OR=1.072 (0.699, 1.645); ER status, OR=1.049 (0.679, 1.619); PR status, OR=1.300 (0.782, 2.161); HER-2 status, OR =1.017 (0.683, 1.516); triple negative breast cancer, OR=0.956 (0.443, 2.063); ki-67 overexpression, OR=1.677 (0.837, 3.362); lymph node status, OR=1.801 (0.991, 3.274); histologic type, OR=1.053 (0.566, 1.960). All the above results could be seen in Table 2.

**Table 2:** Meta-analysis for the association of c-Met overexpression and clinicopathological features of breast cancer patients

Clinicopathological features	No.of studies	No.of patients	Model	OR(95% CI)	*P*-value	Heterogeneity		
						*I*2	*I*2(%)	*P*-Value
Menopausal status (post vs. pre)	3	1210	Fixed	0.76(0.53,1.09)	0.13	1.51	0	0.47
Age(≤50 vs. >50)	4	1438	Random	1.07(0.70,1.65)	0.75	7.6	60.5	0.06
Size(>2cm vs. ≤2cm)	9	2579	Fixed	1.79(1.48,2.15)	0	7.39	0	0.5
ER status(Negative vs. Positive)	11	2718	Random	1.05(0.68,1.62)	0.83	34.62	71.1	0
PR status(Negative vs. Positive)	9	2533	Random	1.30(0.78,2.16)	0.31	29.02	72.4	0
HER-2(Negative vs. Positive)	7	2402	Random	1.02(0.68,1.52)	0.93	13.38	55.1	0.04
TNBC(yes vs. no)	4	2281	Random	0.96(0.44,2.06)	0.91	25.33	88.2	0
Ki67(≥10% vs. <10%)	3	386	Fixed	1.68(0.84,3.36)	0.15	0.66	0	0.72
Histologic grade(G3 vs.G1-2)	14	2418	Random	1.55(1.11,2.16)	0.01	25.08	48.2	0.02
lymph node status(N1-3 vs.N0)	11	2743	Random	1.80(1.00,3.27)	0.05	74.89	86.6	0
Metastasis (yes vs. no)	3	947	Random	33.60(1.64,689.51)	0.02	48.66	95.9	0
Histologic type(IDC vs. ILC)	9	2633	Random	1.05(0.57,1.96)	0.87	15.1	47	0.06

ER: estrogen receptor; PR: progesterone receptor; HER-2: human epidermal growth factor receptor-2; IDC: infiltrating ductal carcinoma; ILC: infiltrating lobular carcinoma.

### Data synthesis: overall survival

**Figure 2:**
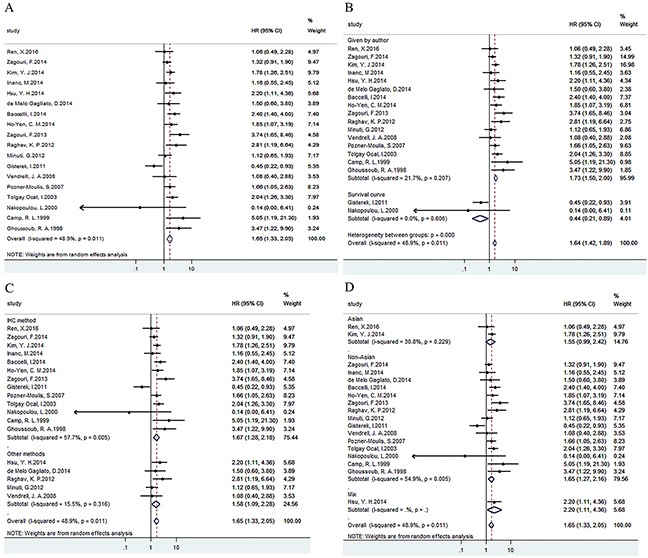
Forest plots of HRs for the association of c-Met overexpression and OS Survival data were reported as OS **(A)**, as well as subgroup analysis of data sources **(B)**, methods **(C)** and ethnicity **(D)** among included studies.

OS was analyzed in 18 studies with 4751 patients. Results showed that c-Met overexpression was related to poor OS, HR=1.65 (1.328, 2.051) (Figure 2A). Besides, results of subgroup analysis according to data sources (Figure 2B)/methods (Figure 2C)/ethnicity (Figure 2D) showed that c-Met overexpression was related to poor OS in Given by author, all methods and all ethnicity groups respectively (Table 3).

**Table 3:** Main meta-analysis results

	Analysis	No.of studies	No.of patients	Model	HR(95% CI)	*P*-value	Heterogeneity		
							*I*2	*I*2(%)	*P*-Value
	**OS**	18	4751	Random	1.65(1.33,2.05)	0	33.24	48.9	0.011
**Data source**	Given by author	16	4380	Fixed	1.75(1.48,2.08)	0	19.15	21.7	0.207
	Survival curve	2	371	Fixed	0.44(0.21,0.89)	0.022	0.27	0	0.606
**Technique**	IHC method	13	4098	Random	1.67(1.28,2.18)	0	28.4	57.7	0.005
	Other methods	5	653	Fixed	1.56(1.12,2.17)	0.009	4.74	15.5	0.316
**Ethnicity**	Asian	2	1051	Fixed	1.63(1.19,2.23)	0.002	1.45	30.8	0.229
	Non-Asian	15	3530	Random	1.65(1.27,2.16)	0	31.04	54.9	0.005
	Mix	1	170	-	2.20(1.11,4.36)	0.024	0	-	-
	**RFS/DFS**	12	3598	Random	1.53(1.20,1.95)	0.001	26.77	58.9	0.005
**Data source**	Given by author	11	2930	Random	1.56(1.19,2.04)	0.001	26.69	62.5	0.003
	Survival curve	1	668	-	1.35(0.87,2.10)	0.182	0	-	-
**Technique**	IHC method	9	2337	Random	1.51(1.11,2.06)	0.008	25.32	68.4	0.001
	Other methods	3	1261	Fixed	1.63(1.17,2.28)	0.004	0.73	0	0.693
**Ethnicity**	Asian	4	1284	Random	1.18(0.64,2.17)	0.59	14.44	79.2	0.002
	Non-Asian	8	2314	Fixed	1.58(1.33,1.87)	0	8.62	18.8	0.281

### Data synthesis: disease/relapse free survival

**Figure 3:**
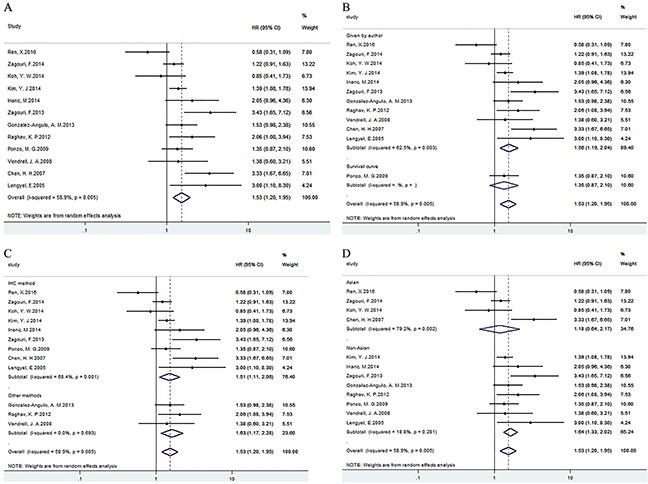
Forest plots of HRs for the association of c-Met overexpression and RFS/DFS Survival data were reported as OS **(A)**, as well as subgroup analysis of data sources **(B)**, methods **(C)** and ethnicity **(D)** among included studies.

Analysis of 12 studies with 3598 patients indicated overexpression of c-Met was related to poor RFS/DFS, HR=1.53(1.20, 1.95) (Figure 3A). Besides, results of subgroup analysis according to data sources (Figure 3B)/methods (Figure 3C)/ethnicity (Figure 3D) showed that c-Met overexpression was related to poor RFS/DFS in Given by author, all methods and non-Asian groups respectively (Table 3).

### Publication bias

**Figure 4:**
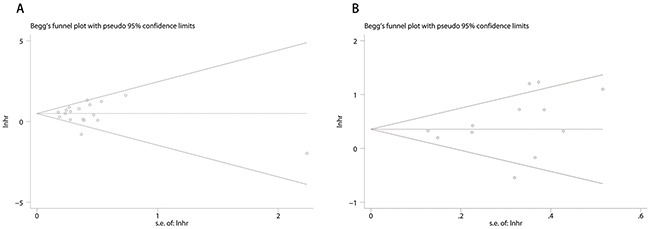
Funnel plots of publication bias of OS and RFS/DFS Publication bias of OS **(A)** and RFS/DFS **(B)** of the meta-analysis showed no statistical signifcance (p > 0.05) using Begg's test.

Funnel plot and Egger’/Begg’ test was used to evaluate publication bias. Results of Egger’/Begg’ test for OS and RFS/DFS were 0.945/0.520 and 0.270/0.131 respectively. Begg's funnel plots with pseudo 95% confidence limits of the OS and RFS/DFS were listed in Figure 4A and 4B.

### Sensitivity analysis

**Figure 5:**
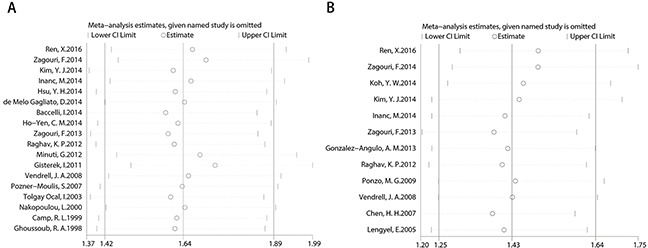
Sensitivity for included studies The effect of single study was evaluated on the whole results of OS **(A)** and RFS/DFS **(B)** in this meta-analysis.

Results of removal of each study at a time could be seen in Figure 5A and 5B. Removal of each study didn't change HR significantly both for the OS and RFS/DFS analysis.

## DISCUSSION

The tyrosine kinase c-Met fosters invasive growth, a complex physiological program that signifies concerted activation of cell proliferation, survival, invasion and angiogenesis [4, 14]. In the past years, mountains of clinical studies have described c-Met overexpression and pathway hyperactivation in tissues of breast cancer patients, and found a strong relationship between high HGF/Met signaling and tumor progression [15, 16]. Our results demonstrated that c-Met overexpression was related to poor OS and RFS/DFS for breast cancer patients. Moreover, c-Met overexpression was associated with large tumor size, high histologic grade and distant metastasis. Therefore, c-Met could be a potential target for breast cancer therapy.

In our meta-analysis, the results of OS showed moderate heterogeneity. Then we conducted subgroup analysis and found that data sources were the origin of heterogeneity. The HR value extracted from survival curve of 2 articles showed a favorable prognosis of c-Met overexpression while other 16 articles with HR value given by author indicated a poor prognosis. The difference is mainly because data extracted from survival curve is not as accurate as that given by author and the article quality is relatively low. Subgroup analysis of RFS/DFS was also conducted on the basis of data source. Only one study with HR value derived from survival curve and both the two subgroups showed poor prognosis of c-Met overexpression. And subgroup analysis of methods reached in same conclusion. Subgroup analysis of ethnicity showed c-Met overexpression in non-Asian group rather than Asian group had statistical difference, which might because the significant heterogeneity in Asian group. What's more, no evidence indicated publication bias for OS and RFS/DFS in regard to c-Met overexpression using Egger’/Begg’ test. And influence analysis of OS and RFS/DFS showed no big difference. All that demonstrated that our results were stable and reliable.

Some studies have investigated the role of c-Met in TNBC and BLBC (basal like breast cancer) and found that c-Met was related to TNBC and BLBC phenotype, which could be exploited as a potential target [2, 9, 17, 18]. Our results showed that c-Met overexpression was independent of hormone receptor status and there was no statistical significance of c-Met overexpression between TNBC and non-TNBC group, which indicated that c-Met could be a target for breast cancer regardless of hormone status. But because of the limited studies, further research is needed to validate the relationship of c-Met overexpression and TNBC/BLBC phenotype.

This study has important implications in breast cancer. Firstly, it demonstrates c-Met overexpression is related to worse OS and RFS/DFS, which indicates that c-Met may be a potential therapeutic target. Secondly, c-Met is involved in malignant biological behavior, such as large tumor size, high histological grade and distant metastasis, and combination therapy with c-Met inhibitor in future will dramatically reduce mortality in invasive breast cancer. However, there are also limitations in this meta-analysis. First of all, identifications of c-Met overexpression of individual studies are not exactly same and as a dichotomous variable, cut-off value may be a source of considerable interstudy heterogeneity. Additionally, although Begg's and Egger's test were performed and there was no statistical significance. Results should be interpreted cautiously because we only include studies with available HR value or K-M survival curves with necessary data.

Currently, the most promising approach for disrupting c-Met signaling is to use small molecular inhibitors to target the intracellular kinase domain [19]. The clinical relevance of c-Met inhibitors is now under investigation, phase II and III clinical trials in a variety of malignancies including non-small cell lung cancer [20–22], colorectal cancer [23], gastroesophageal cancer [24] are ongoing. With regard to breast cancer, a phase II trial examining tivantinib in patients with recurrent or metastatic TNBC [25] and a randomized phase II study evaluating the safety and efficacy of onartuzumab and/or bevacizumab in combination with paclitaxel in patients with metastatic TNBC are currently ongoing [26].

Taken together, our analysis shows that overexpression of c-Met in breast cancer tissues is associated with worse prognosis in human breast cancer. Since c-Met inhibitor has already been investigated in numerous clinical trials, the future clinical application will be easier. Combination therapy of c-Met inhibitor will improve the prognosis of breast cancer patients especially invasive breast cancer and TNBC/BLBC, which are types of the poorest prognosis.

## MATERIALS AND METHODS

### Literature search

This meta-analysis was conducted according to PRISMA guidelines. Studies were identified by searching PubMed and EMBASE databases from 1997 until April, 2016 by using the key words “breast cancer or breast tumor or breast carcinoma” and “hepatocyte growth factor receptor or HGFR or c-Met”. Titles and abstracts were first scanned to exclude irrelevant articles and final inclusion of the articles was determined by reading the full text. The references from identified articles were manually searched for additional relevant records.

### Inclusion and exclusion

All studies in this meta-analysis satisfied the following inclusion criteria: 1) full-text studies published in English; 2) proven diagnosis of breast cancer by pathology; 3) considering the relation between c-Met overexpression and OS, RFS/DFS or clinicopathological features among breast cancer patients; 4) provided the HRs and 95% CIs, or Kaplan-Meier survival curves that provided sufficient data to extract HRs and 95% CIs. Exclusion criteria: 1) no data on survival or clinicopathological features and inability to calculate from Kaplan-Meier survival curve; 2) with previous cancer history.

### Data extraction

Two reviewers (Zhao XX and Qu JK) performed the search and assessed the studies independently. The following items were extracted from each eligible study, including first author, year, patients source, type of patients, protein location, median age, patients number, technique, c-Met overexpression (%), analysis, median follow up, OS/DFS and clinicopathological features. When the univariate and multivariate analysis were both available, the multivariate results were used. If the above-mentioned data was not reported, items should be treated as “NA (not available)”.

### Quality of the studies

The Newcastle-Ottawa Scale was used to assess the quality of each study [27]. The NOS criteria is scored based on three aspects: (1) subject selection, (2) comparability of subject, (3) outcome measurement. NOS scores range from 0 to 9, and a score ≥ 6 indicates a high quality. Two investigators independently assessed the quality of the 32 included studies, and the discrepancies were solved by consensus.

### Statistical analysis

HRs and 95% CIs were used to study the association between c-Met overexpression and OS/DFS. If data were only available in the form of figures, we read Kaplan-Meier curves by Engauge Digitizer version 4.1 (free software downloaded from http://sourceforge.net) and extracted survival data HRs and 95%CI [28]. Data of clinicopathological features was extracted in studies available of ORs. The heterogeneity of included studies was assessed by using I^2^ statistics and P value, and if I^2^ > 50% or P< 0.1, the results were considered statistically significant and random effects models were employed; otherwise, fixed effects models were employed. Sensitivity analysis, also named influence analysis, was carried out to evaluate the effect of single study on the whole results and meanwhile try to find the origin of heterogeneity. Publication bias was assessed graphically using funnel plots, and funnel plot Symmetry was evaluated by Begg's and Egger's linear regression method. P<0.05 was considered statistically significant. Statistical analyses were performed using Stata 13.0 (Stata Corporation, College Station, TX).
